# StandUPTV: a full-factorial optimization trial to reduce sedentary screen time among adults

**DOI:** 10.1186/s12966-025-01771-2

**Published:** 2025-06-13

**Authors:** Sarah K. Keadle, Kristina Hasanaj, Krista S. Leonard, Arlene Fernandez, Lena Freid, Skylar Weiss, Maria Legato, Harsh Anand, Todd A. Hagobian, Siobhan M. Phillips, Suzanne Phelan, Kate Guastaferro, Ryan G. N. Seltzer, Matthew P. Buman

**Affiliations:** 1 Department of Kinesiology and Public Health & Center for Health Research, California Polytechnic State University, San Luis Obispo, CA USA; 2 College of Health Solutions, Arizona State University, Phoenix, AZ USA; 3 Feinberg School of Medicine, Northwestern University, Chicago, IL USA; 4 School of Global Public Health, New York University, New York, NY USA

**Keywords:** Sedentary behavior, Television viewing, Physical activity, Multiphase optimization trial, mHealth application, Screen time

## Abstract

**Background:**

Using the multiphase optimization strategy (MOST) framework, we aimed to identify an optimized mHealth-delivered intervention for reducing recreational sedentary screen time (rSST) by at least 60 min/day among adults.

**Methods:**

Eligible participants were 23–64 years old and self-reported elevated rSST (> 3 h/day). Following a 7-day baseline, participants received a core mHealth application (self-monitoring and 50% reduction target and educational materials) and were randomly assigned to three additional components set to on/off in a full-factorial (2^3^) experiment: LOCKOUT: rSST electronically restricted; TEXT: rSST reduction prompts; and EARN: rSST through physical activity. rSST was assessed at baseline and 16 weeks via an integrated measure that included objectively assessed sedentary time (activPAL accelerometer) and screen time (TV Wifi plugs and tablet usage). We used a linear mixed effect model to evaluate the change in rSST for the three intervention components and their interactions.

**Results:**

A total of 82% of the randomized participants (*N* = 110) were female, with a mean ± SD age of 41 ± 11.7 y and a BMI of 29.7 ± 7.8 kg/m2, and their mean (95% CI) rSST was 184.7 (172.8, 196.5) min/day at baseline. The expected difference (baseline vs. 16 weeks) in rSST was greatest for the intervention versions with the core plus EARN on with an average reduction of -118.1 (-163.0, -73.1) min/day and for core plus LOCKOUT, TEXT, & EARN on (-125.7 [-172.0, -79.3] min/day).

**Conclusions:**

We identified several promising intervention versions that exceeded our optimization objective. This study provides important evidence on efficacious multicomponent interventions that should be moved forward to the evaluation phase of the MOST framework to test the effect of rSST reductions on health outcomes.

**Trial registration:**

(clinicaltrials.gov NCT04464993)

**Supplementary Information:**

The online version contains supplementary material available at 10.1186/s12966-025-01771-2.

## Background

Television viewing (TV) consumes ~ 55% of discretionary time, an average of over 3 h/day [1, 2]. The widespread adoption of social media, video games, and streaming services that can be accessed via smartphones and tablets has resulted in alarming net increases in total recreational sedentary screen time (rSST) [1–4]. This increasing trend was exacerbated by the COVID-19 pandemic [4–6]. There are consistent and robust associations between prolonged TV viewing and numerous poor health outcomes, including diabetes, cardiovascular disease, mental health, and mortality [7–12]. TV viewing is associated with eight of the leading causes of death in America, with increased risk at 3–4 h/day for most mortality outcomes [9]. Modern methods of consuming rSST promote ‘binge viewing’ (i.e., watching consecutive episodes in a continuous bout) [13], which is particularly harmful to health [14]. The risk of poor health is greater for TV viewing than for non-screen based sedentary behaviors and the negative effects of TV viewing are not fully eliminated through engaging in recommended amounts of physical activity [15–17]. Emerging evidence also suggests that non-TV forms of rSST (e.g., social media and video games) are also associated with poor sleep quality, mood, and less physical activity [18–20]. 

Given the high prevalence and the known health risks, reducing rSST is an important target for public health, yet few intervention studies have been conducted among adults [21, 22]. In 2016, the Community Preventive Services Task Force reviewed behavioral strategies to reduce screen time in adults [21] and identified just two studies among adults [23, 24]. Since that time, we are aware of one additional intervention that targeted screen time as a weight loss strategy among adults [25]. Collectively, the available studies reported significant reductions in SSTs of > 60 min/d but were limited by small samples (*N* < 40), short durations (3–8 weeks), and only traditional TV viewing. Furthermore, no study has compared the combined effect of multiple strategies to reduce SST, and none has adapted to contemporary rSST consumption (i.e., social media, tablets, streaming media), which precludes recommendations on optimal intervention strategies to reduce rSST for adults [21]. 

To address this gap, we applied the multiphase optimization strategy (MOST) framework, which has three phases (preparation, optimization and evaluation) and is designed to efficiently and systematically develop and evaluate multi-component interventions [26]. In the preparation phase [27], we identified three candidate components and established an optimization objective of at least a 60 min/d reduction in rSST, based upon previous work demonstrating clinically significant improvement in chronic disease biomarkers [28]. The goal of the optimization phase is to enable researchers to evaluate which intervention component (i.e., a distinct intervention strategy or delivery method) - independently or in combination- meaningfully contribute to the desired outcome [26]. The purpose of our optimization study was to identify a multicomponent intervention that would achieve a reduction in the rSST of at least 60 min at 16-weeks post randomization while considering the user burden and acceptability of the intervention package (i.e., all active components optimization objective). We conducted a 16-week 2^3^ full- factorial randomized trial that evaluated the effects of the three candidate components, alone and in combination, on total rSST. We also present the results for secondary outcomes of total sedentary time, light physical activity (LPA), and moderate-vigorous physical activity (MVPA).

## Methods

### Participants

The trial was registered at clinicaltrials.gov (NCT04464993) and the full study protocol was published previously [27]. Data collection was scheduled to begin in July 2020 but was delayed until May 2021 because of COVID-19. Specifically, due to COVID-19 we removed planned cardiovascular biomarker assessment and modified the protocol to conduct all study visits virtually, which opened recruitment to those outside the Phoenix, AZ and San Luis Obispo, CA areas. Enrollment continued until April 2023, and data collection was completed in September 2023. Recruitment methods included newspaper advertisements, paper and electronic flyers sent to the university and local community, ResearchMatch.com, social media (e.g., Facebook, Instagram).

Eligible participants were between 23 and 64 years of age (i.e., working aged-adults with high rSST levels and heterogeneous media consumption) [29]. Enrollment and randomization were stratified by age group (23- 44.9 y vs. 45- 63.9 y). The eligibility criteria were described in detail previously [27]. Briefly, participants self-reported > 3 h/day recreational sedentary screen time, < 3 on the Stanford Leisure-Time Activity Categorical Item (L-Cat) [30] had no medical contraindications for MVPA, had smartphone and WiFi access and were willing to comply with the study procedures. In response to difficulties with recruitment during the COVID-19 pandemic and because in-person visits with biomarkers were removed from the protocol, we lowered the BMI threshold to include those with a BMI *≥* 18.5 kg/m2 on April 11, 2022. The screening forms were completed via Research Electronic Data Capture (REDCap). Eligible individuals received an email with the online version of the informed consent document that was approved by the Arizona State University Institutional Review Board (IRB #00012109) and scheduled a video conferencing visit with staff who explained the study procedures and expectations of the study participants. Eligible and interested participants electronically signed the informed consent document online via REDCap [31, 32]. 

### Enrollment and baseline procedures

After providing consent, the participants were mailed via U.S. Postal Service a technology kit that included several devices for tracking rSST: a Wi-Fi plug per TV in the home (WeMo Insight Smart Plug); one Wi-Fi router; one Raspberry Pi (i.e., a small computer to record and transmit data); one Samsung Galaxy tablet preloaded with apps that participants stated they regularly used on their smartphones; and a Fitbit Charge 4. Participants were encouraged to use the tablet for all screen time that was not consumed on their home TV(s) including any rSST apps on their smartphone. Baseline assessments were performed over 7 days. All screen time was monitored, and participants were able to use the study tablet but did not yet have access to intervention content or feedback on rSST. The participants were required to have ≥ 4 days of measured screen time (i.e., verification from the study server that tablet and WeMo data were transmitted > 10 h/day) during the baseline period and were asked to repeat the baseline test if they had < 4 days recorded.

### Randomization and intervention

The (2^3^) full-factorial design represented all combinations of the three candidate components yielding eight experimental conditions to which participants were randomly assigned (Table 1). Randomization was stratified by age category (23.0 to 44.9y vs. 45.0 to 64.9y) and conducted using the MOST randomization module in RedCap [33] Following the baseline assessment, participants attended a virtual visit with a staff member where they were notified of their experimental condition, reviewed their motivations for joining the study and reasons for wanting to reduce their rSST. They also received a brief orientation to the app that included reviewing one educational lesson of their choosing and demonstrating how to navigate the app, including features specific to the components in their assigned experimental condition. Investigators were blinded to intervention assignment.

**Table 1:** Experimental design

Experimental condition	CORE	TEXT	LOCKOUT	EARN
1	ON	ON	ON	ON
2	ON	ON	ON	OFF
3	ON	ON	OFF	ON
4	ON	ON	OFF	OFF
5	ON	OFF	ON	ON
6	ON	OFF	ON	OFF
7	ON	OFF	OFF	ON
8	ON	OFF	OFF	OFF

Note: Core included educational materials, 50% reduction target for recreational sedentary screen time (rSST), and self-monitoring; LOCKOUT: rSST electronically restricted; TEXT: rSST reduction prompts; and EARN: rSST through physical activity

### Intervention

The component selection, conceptual model and intervention details were previously published and are briefly described below [27]. Supplemental Table 1 shows the behavior change techniques within the core intervention and by component, according to Michie et al., (2013) behavior change technique taxonomy [33]. All participants received a core intervention via the StandUPTV mHealth application that included (a) a target to reduce screen time by 50% compared with their baseline values; (b) rSST self-monitoring tools (i.e., the gauge, graphs and charts); and (c) 16 lessons, including education and behavioral change content. The 50% reduction target was chosen to be consistent with previous studies that achieved significant television time reduction [23, 24]. rSST was monitored continuously throughout the intervention using minute-level fitbit data (extract in near real-time through Fitbit’s application programming interface [API]) combined with the WiFi plug (TV-time) and tablet data. Technical details for screen time integration are below.

*LOCKOUT.* Screen-limiting features were enabled for both the traditional TV (via the WiFi plug) and the recreational apps on the tablet when rSST reached the prescribed threshold (50% of baseline) within a week-long period. The 50% baseline allotment was reinstated at the beginning of the next week. Participants were granted one “mulligan” per week that enabled them to delay a lockout by 60 min (e.g., to finish a show). Participants also received a planning tool interface for scheduling screen time up to one week in advance.

*TEXT.* Participants with the TEXT condition on received between 1 and 3 prompts per day, which included (a) encouraging use of the intervention strategies; (b) 7-day rSST summaries; (c) summaries of specific rSST behaviors (e.g., “You have watched X hours of screen time over the last 7 days in bouts of 1 hour or longer”); and (d) encouraging prompts regarding rSST and (e) adaptive contextual messages on the basis of time of day and screen time (e.g., “Time to hit pause on screen time! Get up and dive into something new!”).

*EARN.* Participants were able to earn screen time by engaging in Fitbit-assessed MVPA in 10-minute bouts at a ratio of 1:3 (i.e., 10-min exercise earns 30-min rSST). Those with EARN and LOCKOUT were able to earn more screen time to avoid LOCKOUT. The gauge display was modified for this component to emphasize the overall weekly rSST goal, time spent in MVPA, and earning balance (i.e., the difference between total screen time and amount earned). They also received six additional educational lessons regarding MVPA (e.g., safe exercising, barriers to exercise) and an interactive interface to plan MVPA time.

### Measures

The assessment timepoints were baseline, mid-intervention (8 weeks), and post-intervention (16 weeks). This paper focuses only on 16-week outcomes to remain consistent with our optimization objective.

*Primary outcome*: rSST was assessed using a time-synchronized combination of accelerometer-measured sedentary time (activPAL) and screen time data from the StandUPTV mHealth application. The technical details have been previously published and are briefly described below [27]. 

*Posture and activity intensity* were assessed using the activPAL3c micro accelerometer (PAL Technologies Ltd, Glasgow, Scotland). The devices were waterproofed using a medical grade adhesive covering and attached to the midline of the thigh with breathable, hypoallergenic tape, enabling continuous wear for consecutive days without removing for bathing or other water-based activities. Participants were asked to wear the device 24 h a day for 7 days, and the following periods were excluded: (a) continuous sitting or standing behavior > 6 h (considered nonwear); (b) days with ≤ 10 h of valid wear time during the wake period; and (c) participants with only 3 or fewer valid days of activPAL wear. The data were processed into events of sitting/lying, standing, or stepping using the activPAL software version 7.2.37. All wake time measured by the activPAL as lying/seated was categorized as sedentary. Stepping time was split into periods of light-intensity physical activity ([LPA]; <100 steps/minute) and MVPA (≥ 100 steps/minute) [27]. 

*Screen time* was assessed via a combination of direct measurement from WiFi Plugs to monitor television power state (Wemo Insight, Belkin, El Segundo, CA) and tablet app usage (Samsung Galaxy, Samsung). All television was considered recreational. The study staff classified more than 730 tablet apps into categories such as video games, television/video, social media, or non-recreational.

The three streams of data (TV, tablet, activPAL) were merged at the minute level for the primary determination of rSST. To label a minute as rSST, it was required to be both sedentary by the activPAL and recreational screen time by either the tablet or television. Users were able to add or modify screen time bouts through the app when screen time occurred outside of the home or on alternative devices or reject bouts when another household member was viewing the screen.

*Feasibility* included metrics of demand and implementation and was assessed by examining study recruitment and retention metrics, including formal withdrawal from the study and completed assessments at 16 weeks [34]. 

*Acceptability* outcomes focused on assessing how the individual recipients reacted to the intervention and we focused on whether it varied substantially by component [34]. After the 16-week assessment, participants rated overall satisfaction with the StandUPTV app (i.e., 5-item Likert scale), how often they used it, whether they would continue to use the app or recommend it to others, and whether it was helpful in reducing rSST. We also examined satisfaction with staff and technical support. Additional questions were asked that were relevant to specific components (e.g., those with TEXT on were asked to rate how helpful the text messages were in changing behavior). The response options were scored from 0 to 10, with 0–2 considered “not at all”, 3–7 considered “mostly” and 8–10 considered “very”. In addition, qualitative exit interviews were conducted among all participants via a series of open-ended questions. User burden was assessed on the User Burden Scale, a 20-item scale that measures burden across six domains (difficulty of use, physical, time/social, mental/emotional, privacy, financial) [35]. 

### Sample size and statistical analysis

We aimed to enroll 240 participants to achieve a final sample of 200 (20% dropout; *n* = 25 per condition), which afforded power = 0.80 to detect main effects for each of the intervention components (LOCKOUT, TEXT, and EARN) and the resulting 2- and 3-way interactions, assuming a balanced experimental design and a modest baseline-to-postintervention correlation (*r* = 0.3) and α = 0.05 [36]. We powered the study on a Cohen’s d effect size = 0.4, representing a 60 min/day reduction in sedentary behavior. This effect size was conservative, as previous rSST trials reported effects d > 1.0 [23, 24]. Owing to delays in initiating and slowing recruitment during the COVID-19 pandemic, we did not meet the enrollment targets, and the trial concluded when funding ended.

Multiple imputation using chained equations (MICE) was employed to address missing data due to dropout, nonresponse, or incomplete data collection using the MI procedure. Randomness of missing data was evaluated with a generalized linear mixed model in which model predictor variables were regressed on a missing data indicator variable. While some model variables were significantly related to the missingness outcome, rejecting the hypothesis of missing completely at random, other model variables were not statistically significant, providing evidence that the missing data may be missing at random, and therefore appropriate for MICE methods. The imputation process was performed in 25 iterations using time, the three study groups of EARN, TEXT, and LOCKOUT, rSST, sedentary time, LPA and MVPA (min/d) [37, 38]. Imputation used both linear and logistic regressions for continuous and categorical data, respectively.

After imputation, the analyses were conducted using the pooled estimates from the multiple imputed datasets. We used a linear mixed model (proc MIXED) to test hypotheses regarding the main effects of three candidate intervention components and their interactions on the primary outcome, rSST. Effect coding was where components were coded as 1 when on and − 1 when off, and we evaluated all single component (e.g., LOCKOUT*time) two- and three-way interactions (e.g., LOCKOUT*EARN*TEXT*time) within the model. Notably, in the full-factorial design, conditions are assigned orthogonally, and estimation of each main effect and interaction effect utilizes data contributed by all the subjects in the trial. For example, the interpretation of the intervention version (i.e., intervention component combinations) that includes CORE & TEXT is a comparison of experimental conditions 1, 2, 3, and 4 (where TEXT was “on”) versus experimental conditions 5, 6, 7, and 8 (where TEXT was “off”) (Table 1). The least squared means, standard errors, and confidence intervals were calculated and pooled across the 25 imputed datasets via Rubin’s rules with the MIANALYZE procedure. The model variables of time, EARN, TEXT, and LOCKOUT were by groups for this procedure [36]. Effect size (Cohen’s d) was calculated by dividing the difference in least squared means between baseline and 16-weeks by the pooled standard deviation [39]. These analyses, including imputation procedures, were repeated for secondary outcomes. All analyses were conducted in SAS 9.4.

The quantitative data acceptability and user burden scale were analyzed via descriptive statistics (e.g., means, standard deviations, frequency). The user burden responses were categorized based on percentile scores as low burden (16th–35th), moderate burden (36th–85th), moderately-high burden (86th–94th) and high burden (≥ 95th) [35]. Due to a focused scope on acceptability and enhancing future iterations of StandUPTV, responses during the final exit interviews were summarized using a rapid qualitative analysis (RQA) approach [40]. A brief interview guide was developed by the research team to elucidate salient themes of likes and dislikes of the StandUPTV app, and suggestions for improvement. Detailed field notes were taken during each exit interview by research staff (ML and AF) and summaries from each participant were provided to the research team. Two trained students and principal investigators (SK and MB) reviewed the field notes and identified themes of likes, dislikes, and suggestions for improvement.

## Results

**Fig. 1:**
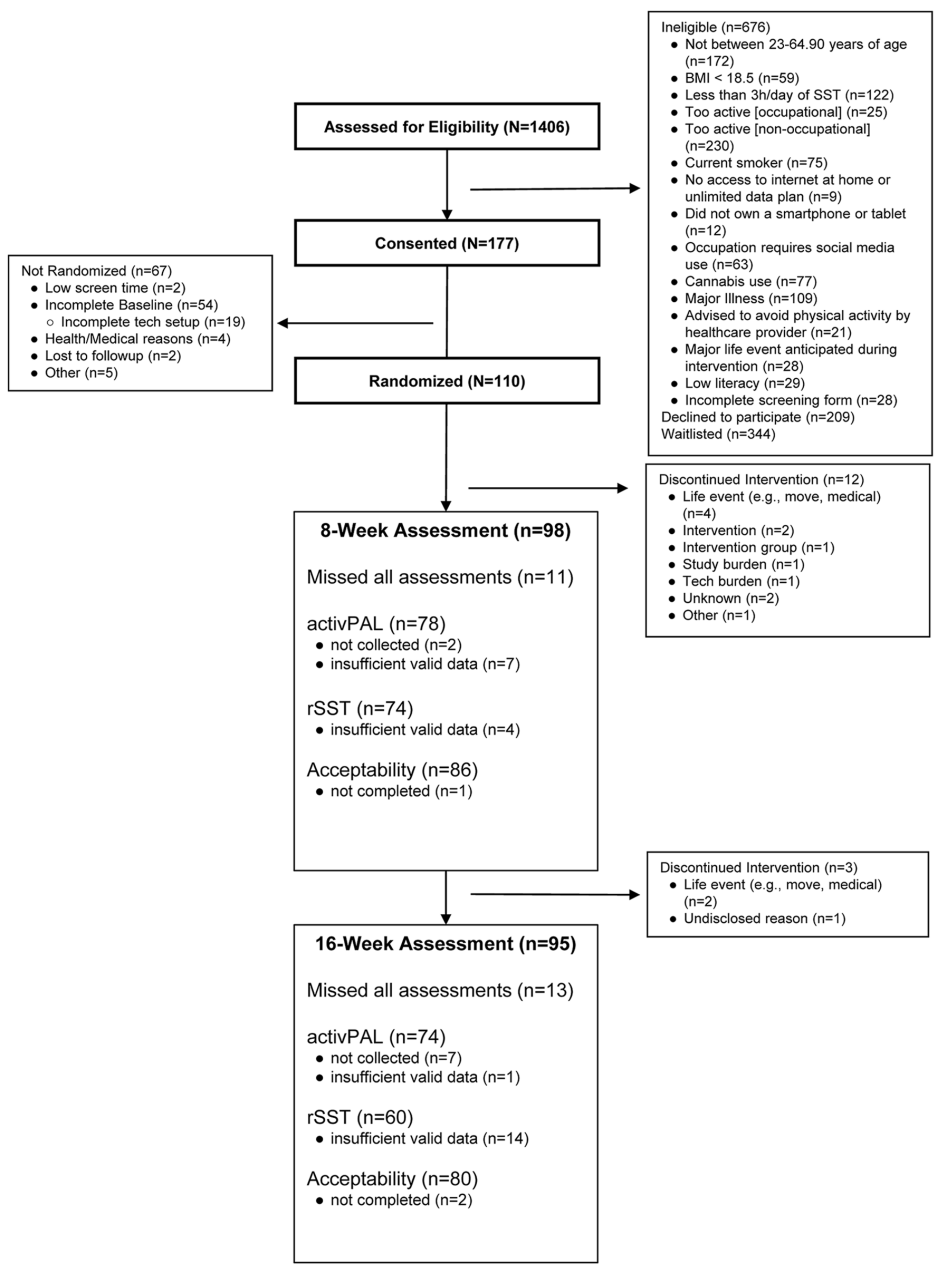
Consort Diagram illustrating flow of study participants

Figure 1 presents the participant flow for recruitment, randomization, and retention. In total, 1406 individuals were assessed for eligibility; 177 consented, and 110 were randomized. Of the 1406 screened, 521 (37%) were through social media and 434 (31%) were from university advertisements and announcements. A total of 95 participants completed the 16-week intervention (86% retention), with the majority of those who discontinued the intervention doing so within the first 8 weeks and due to life events (e.g., relocation or medical). The participant demographics and baseline study outcomes are presented in Table 2. The sample was predominantly female, middle-aged, overweight, and non-Hispanic White (22% Hispanic). At baseline, participants engaged in > 3 h of rSST (measured objectively via activPAL and the screen-time measurement platform) and > 10 h of total sedentary time (measured via activPAL) per day. There were no appreciable differences in baseline characteristics or outcome measures at the component level or among those who completed vs. did not complete the study (Supplemental Tables 2 and 3).

**Table 2:** Baseline demographics characteristics of the randomized sample

Sample size		*n* = 110	
**Female**	**No. (%)**	90 (81.8%)	
**Age (yrs)**	**Mean (SD)**	42.0 (11.7)	
**BMI (kg/m2)**	**Mean (SD)**	29.7 (7.8)	
**Race**,** N (%)**	**Caucasian**	82 (74.6%)	
	**African American**	5 (4.6%)	
	**Asian**	14 (12.7%)	
	**Other**	12 (10.9%)	
**Ethnicity**,** N (%)**	**Non-Hispanic**	87 (79.%)	
	**Hispanic**	23 (20.9%)	
**Outcome variables**,** Mean (SD)**			
**rSST**	**min/day**	184.1 (125.8)	
**Total sedentary time**	**min/day**	641.4 (98.8)	
**LPA**	**min/day**	71.2 (15.5)	
**MVPA**	**min/day**	20.6 (14.8)	
**Steps**	**Steps/day**	6736 (2816)	

Note: rSST is recreational sedentary screen time, Sedentary time, Light physical activity and moderate physical activity were all assessed using the activPAL

### Intervention impact on rSST at 16 weeks

**Fig. 2:**
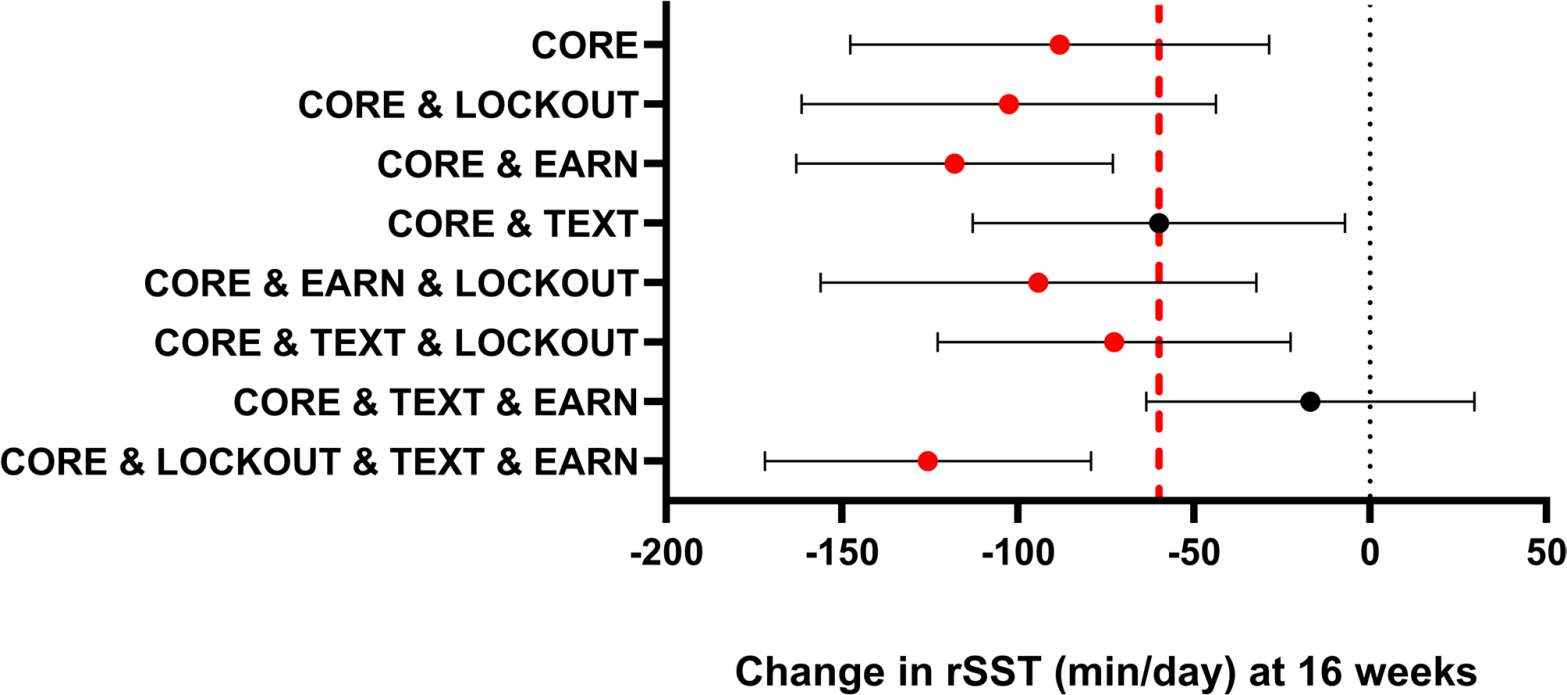
Expected outcomes (Change in rSST) by Intervention Version. Note: Values are min/day

At 16 weeks, 75% of the participants provided data for analysis. The rSST and overall sedentary time outcome data were missing, primarily because of insufficient valid data from the activPAL or technical errors in the screen-time monitoring platform. There was an overall significant main effect of time, as participants engaged in a mean (95% CI) of 184.7 (172.8, 196.5) min/day of rSST at baseline, which was reduced to 99.8 (85.6, 114.0) min/day at week 16. The expected difference (baseline vs. 16 weeks) in the primary optimization outcome (rSST) is shown in Fig. 2 for each intervention version and in Table 3 for the mean at each time point. The expected difference exceeded the a priori identified optimization objective of 60 min/day for 6 of the 8 intervention versions. The two intervention versions with the greatest expected differences were EARN & TEXT & LOCKOUT (-125.7 [-172.0, -79.3] min/day, *d*=-0.73) and EARN (-118.1 [-163.0, -73.1] min/day, *d*= -0.71). The additional intervention versions exceeding 60 min/day were LOCKOUT (-102.7 [-161.5, -43.8] min/day, *d*=-0.5), TEXT & LOCKOUT (-72.8 [-122.8, -22.6] min/day, *d*=-0.39), and EARN & LOCKOUT (-94.3 [-156.2, -32.4) min/day, *d*=-0.41]. TEXT met but did not exceed the optimization objective (-60.1 [-113.0, -28.8] min/day, *d*=-0.31). The only intervention version with some indication of antagonism among components was TEXT & EARN, with a reduction in rSST of -15.9 (-23.8, -8.0) min/day and the smallest effect size (d=-0.10).

**Table 3:** Changes in rSST, total sedentary time light, and moderate-vigorous physical activity by intervention version

	rSST (min/day				Sedentary Time (min/day)			
	Baseline	Week 16	Difference	Cohen’s d	Baseline	Week 16	Difference	Cohen’s d
**OVERALL**	184.7 (6.1)	99.8 (7.3)	**-84.8 (-103.6**,** -66.1)**	-1.24	635.8 (5.7)	635.3 (7.4)	-0.4 (-18.7, 17.8)	-0.01
**LOCKOUT&EARN&TEXT**	194.1 (16.1)	68.5 (17.2)	**-125.7 (-172.0**,** -79.3)**	-0.73	672.7 (14.5)	674.8 (15.9)	2.1 (-40.0, 44.3)	0.01
**LOCKOUT&EARN**	210.0 (20.3)	115.7 (24.2)	**-94.3 (-156.2**,** -32.4)**	-0.41	634.9 (18.9)	691.3 (26.6)	56.4 (-4.8, 117.5)	0.24
**LOCKOUT&TEXT**	178.1 (16.1)	105.3 (19.9)	**-72.8 (-122.9**,** -22.6)**	-0.39	633.6 (14.8)	635.2 (18.7)	1.5 (-45.2, 48.3)	0.01
**LOCKOUT**	175.3 (19.0)	72.6 (21.2)	**-102.7 (-161.5**,** -43.8)**	-0.50	608.9 (17.9)	636.8 (19.6)	27.9 (-22.9, 78.7)	0.15
**TEX&EARN**	118.9 (15.9)	101.9 (17.7)	-17.0 (-63.6, 29.6)	-0.10	628.2 (14.6)	613.3 (16.5)	-14.9 (-58.2, 28.4)	-0.09
**EARN**	240.9 (14.9)	122.9 (17.4)	**-118.1 (-163.0**,** -73.1)**	-0.71	615.1 (13.7)	606.9 (15.8)	-8.2 (-49.2, 32.8)	-0.05
**TEXT**	143.0 (16.5)	82.9 (21.3)	**-60.1 (-113.0**,** -7.2)**	-0.31	622.3 (15.2)	583.7 (19.7)	-38.6 (-87.4, 10.3)	-0.22
**CORE ONLY**	216.9 (18.7)	128.6 (23.4)	**-88.2 (-147.7**,** -28.8)**	-0.41	670.6 (17.3)	640.9 (22.4)	-29.7 (-84.5, 25.1)	-0.15

	**Light Intensity Physical Activity (min/day)**				**Moderate-Vigorous Physical Activity (min/day)**			
	Baseline	Week 16	Difference	Cohen’s d	Baseline	Week 16	Difference	Cohen’s d
**OVERALL**	71.3 (1.5)	73.1 (1.9)	1.9 (-3.0, 6.7)	-0.11	18.6 (0.8)	16.1 (1.1)	-2.5 (-5.1, 0.2)	-0.26
**LOCKOUT&EARN&TEXT**	63.4 (4.0)	67.7 (4.3)	4.3 (-7.2, 15.8)	-0.10	14.2 (2.1)	14.4 (2.2)	0.3 (-5.8, 6.3)	0.01
**LOCKOUT&EARN**	75.6 (5.1)	69.8 (6.1)	-5.7 (-21.9, 10.4)	0.10	8.6 (2.6)	10.0 (3.9)	1.5 (-7.6, 10.6)	0.04
**LOCKOUT&TEXT**	63.5 (4.0)	66.9 (5.1)	3.4 (-9.3, 16.1)	-0.07	32.1 (2.1)	16.6 (2.8)	-15.6 (-22.4, -8.8)	-0.63
**LOCKOUT**	79.5 (4.5)	78.0 (5.2)	-1.5 (-15.1, 12.1)	0.03	14.6 (2.5)	15.6 (2.9)	1.0 (-6.2, 8.2)	0.04
**TEX&EARN**	71.9 (4.0)	77.0 (4.3)	5.1 (-6.4, 16.6)	-0.12	22.6 (15.9)	23.8 (2.3)	1.2 (-4.9, 7.3)	-0.03
**EARN**	81.9 (3.7)	80.3 (4.1)	-1.6 (-12.5, 9.3)	0.04	19.3 (1.9)	18.6 (2.3)	-0.7 (-6.7, 5.2)	0.05
**TEXT**	73.0 (4.1)	76.4 (5.4)	3.4 (-9.9, 16.6)	-0.07	16.2 (2.2)	16.1 (2.9)	-0.1 (-7.2, 6.9)	0.00
**CORE ONLY**	61.3 (4.7)	68.8 (6.2)	7.5 (-8.3, 23.3)	0.13	21.1 (2.5)	13.9 (3.2)	-7.2 (-14.8, 0.4)	0.01

Note: baseline and week 16 values are mean (SE) from mixed model. Differences are mean (95% confidence interval). rSST is recreational sedentary screen time, Sedentary time, Light physical activity and moderate physical activity were all assessed using the activPAL. Cohen’s d is the mean difference at baseline and 16 weeks divided by pooled standard deviation. Bold indicates statistical significance (*p* < 0.05)

### Secondary outcomes: impact on sedentary time and physical activity

There was no significant reduction in total sedentary time at 16 weeks (Table 3) and no evidence that any component led to reductions in total sedentary time. Overall, there was no significant increase in LPA or MVPA in any of the intervention versions.

### Study feasibility, user burden, and acceptability

Table 4 shows the feasibility, acceptability and user burden overall and by component. Among the 110 randomized participants, 11 withdrew (10%) for reasons related to the study (e.g., burden), an additional 5 (4.5%) withdrew for unrelated reasons (1 moved out of the country, 1 moved and started school, 2 reported unrelated injuries, 1 no longer had a stable living situation), and 14 were unresponsive at the 16-week assessment (12.7%). This resulted in 80/110 (72.7%) randomized participants completing the 16-week assessment. Two harms were reported that were deemed unrelated to the intervention: severe concussion and undisclosed injury. Both participants withdrew. One participant withdrew due to reported mild skin irritation caused by Fitbit. Retention was slightly higher when LOCKOUT was off vs. on (76.4% vs. 69.1%) and higher when EARN was on (77.2%) vs. off (69.8%). Supplemental Table 4 shows the same results across the experimental conditions; retention was highest among LOCKOUT & TEXT and EARN & TEXT & EARN (> 85%) and lowest among LOCKOUT & EARN and LOCKOUT & TEXT (< 55%), but the given small cells should be interpreted with caution.

**Table 4:** Feasibility, acceptability and user burden outcomes overall and by component

		Overall	LOCKOUT		TEXT		EARN	
			On	Off	On	Off	On	Off
**Feasibility**	Randomized (N)	110	55	55	59	51	57	53
	Study-related withdraw (N[%])	11 (10%)	7 (12.7%)	4 (7.3%)	6 (10.2%)	5 (9.8%)	2 (3.5%)	8 (15.1%)
	Non-study related withdraw (N[%])	5 (4.5%)	1 (1.8%)	4 (7.3%)	2 (3.4%)	3 (5.9%)	3 (5.3%)	2 (3.8%)
	Missing 16-week assessment (N[%])	14 (12.7%)	9 (16.4%)	5 (9.1%)	7 (11.9%)	7 (13.7%)	8 (14.0%)	6 (11.3%)
	Completed 16-week assessment (N[%])	80 (72.8%)	38 (69.1%)	42 (76.4.%)	44 (74.6%)	36 (70.6%)	44 (77.2%)	37 (69.8%)
**Acceptability**		**% satisfied/very satisfied or % yes**						
Overall Satisfaction with StandupTV app (% satisfied)		55	57	53	49	61	51	59
Overall, was the StandUPTV app helpful in reducing your recreational sedentary screen time? (% yes)		94	100	90.0	90	97	89	97
If it were made available, would you be interested in continuing the use of the StandUPTV app after the study is over? (% yes)		78	74	81	81	75	77	79
Would you download an app like this to your smartphone? (% yes)		85	78	90.0	84	86	81	89
Would you recommend the StandUPTV app to other people? (% yes)		92	92	93	90	94	89	96
Proportion using app at least 1/day (% yes)		59	70	51	65	55	61	57
Satisfaction with technical support		85	75	92	85	85	89	79
Responsiveness of study staff		99	100	97	100	97	97	100
**User burden** (**Total Score**,** M[SD])**		7.2 (6.5)	7.6 (6.3)	6.8 (6.8)	8.1 (6.6)	6.1 (6.4)	7.9 (6.3)	6.3 (6.8)
		*N* = 79	*N* = 37	*N* = 42	*N* = 43	*N* = 36	*N* = 44	*N* = 35

The acceptability scores are shown in Table 4. Overall, and across components, participants were very satisfied with the study staff (> 98.5% satisfied or very satisfied). They were satisfied with technical support from staff (84.8%), although satisfaction was slightly lower when LOCKOUT was on (74.6%) than when LOCKOUT was off (92.1%). The mean (SD) user burden score was 7.2 [6.5] on a scale ranging from 0 to 80. The highest subdomain score was for *difficulty of use*, which was 0.8 (0.6) and was considered a moderate burden. *The scores for the time*,* social*, *mental*,* emotional* and *privacy* domains were all somewhat low, and the scores for the *financial* and *physical* domains were very low. Burden scores did not vary by condition. (Supplemental Table 4).

In response to the question “rate your satisfaction with the StandUPTV app,” 54.5% reported being somewhat or very satisfied, 25.6% were neutral, and 19.7% were somewhat or very dissatisfied. Interestingly, despite modest overall satisfaction, a high proportion said that StandUPTV was helpful in reducing rSST (93.4%), that they would recommend the app to other people (91.8%), would download a similar app to their smartphone (83.9%) and would be interested in continuing to use the app after the study was over (78.7%). The majority (60.7%) used the app at least daily. The quantitative responses for the component-specific questions are shown in Supplemental Table 5. When TEXT was on, a greater proportion of the participants were not satisfied with the app (18.8%), and the majority (53%) did not find the text messages helpful. As noted in Supplemental Table 6 with the qualitative themes, several people reported not receiving or missing notifications. Some had issues with multiple notifications appearing at once and reported swiping them all away. Feedback on the content and frequency of messages was evenly distributed among those who found it not at all, mostly and very helpful. Those who liked the messages responded to the personalized messages in response to rSST and those that they perceived as tailored to their interests. The majority of the EARN participants stated that the opportunity to earn screen time through exercise was very helpful (69.4%). They were more satisfied with the feedback on exercise (55.6% very satisfied) than with the feedback on the planning tool (11.1% very satisfied). The majority reported that the earnings ratio was adequate or too high, and several stated that it could be lowered to 1:2.

The qualitative feedback is summarized in Supplemental Table 6. In response to the question “What did you like least about the StandUPTV app?”, the feedback was primarily related to the tablet and the bout verification process used to authorize screen time. The most well-liked feature was the self-monitoring gauge that tracked weekly screen time, which participants reported increased their self-awareness of their rSST and was a simple way to track screen time across platforms. They noted increased awareness of their total amount of free time and how much of that was consumed by screens.

## Discussion

Several intervention versions exceeded the 60 min/day optimization objective, all combining CORE with components including LOCKOUT, EARN, EARN & LOCKOUT, TEXT & LOCKOUT, and EARN, TEXT & LOCKOUT. The CORE intervention included self-monitoring, a 50% rSST reduction goal, and 16 educational lessons. The most promising intervention versions were EARN alone and EARN, LOCKOUT & TEXT, both showing similar acceptability and user burden but with distinct trade-offs. EARN is simpler and does not require programming time for implementing the other two components. In contrast, the multi-component version (EARN, LOCKOUT & TEXT) offered slightly better outcomes without added user burden and includes behavioral consequences for exceeding rSST targets and timely messaging (TEXT)—both effective elements in behavior change. Both intervention versions yielded an optimized intervention that could be evaluated in future studies.

Our results are similar in magnitude to previous studies. Otten et al. reported a > 2 h/day reduction in self-reported SST using an electronic “lockout” method where participants received a target to reduce TV by 50% and when the limit was reached the television shut off until the following week [23]. Similarly, Raynor et al. demonstrated a > 2 h/day reduction in self-reported SST by instructing adults to gradually decrease TV watching via behavioral strategies such as stimulus control (e.g., removing remote control) and preplanning in combination with electronic monitoring of television (but not lockout) during a 3-week intervention period [24]. Our two intervention versions with the greatest expected difference also produced a difference of approximately 2 h/day.

Reductions in rSST did not have substantive spillover effects on exercise behaviors, which is also consistent with previous research [24]. Raynor et al., [24] revealed that a “decrease TV” study arm did not result in increases physical activity, but a study arm that received both “decrease TV plus increase PA” did show improvements in steps and MVPA. In our study, design to optimize on rSST, we purposely did not include physical activity promotion materials in the CORE and suggested a range of “alternative activities” that include both physical activities and sedentary behaviors. We found that, on average, participants appeared to replace rSST with non-rSST sedentary behaviors rather than physical activities. In the present study, those with EARN “on” did receive educational content (6 lessons) and self-monitoring of MVPA, yet the results for MVPA changes were equivocal across intervention versions. Although there may be health benefits for replacing rSST with other sedentary behaviors, given the known benefits of physical activity, it would be ideal to leverage reductions in rSST into increases in physical activity [41]. Future research should examine the displacement effects of rSST on specific activity domains and intensities and behaviors to inform interventions strategies to effectively both reduce rSST and increase physical activity.

Compared to the core component, the TEXT component did not consistently reduce rSST and was even counterproductive when combined with EARN—an unexpected finding given that text messages often promote physical activity [42]. Technical and conceptual issues likely contributed to its limited effectiveness. Messages were delivered as app notifications on tablets, not SMS on smartphones, and some participants reported frustration with receiving multiple messages at once or difficulty accessing older messages. Prior research shows that poorly timed or “irrelevant” messages negatively impact physical activity [43]. In this study, many messages were time-sensitive or triggered by rSST duration, so technical delays may have undermined their relevance and increases dissatisfaction with TEXT. The MOST framework allows for iterative refinement [26]. Future research should address these technical issues by to ensure messages are timely and relevant—key to effective health messaging.

This study had limitations and offered valuable lessons to improve mHealth app acceptability and effectiveness for reducing rSST in future iterations. Using a study tablet lowered participant satisfaction and fidelity; participants consistently preferred the idea of having the app on their own smartphones. Because the app wasn’t available on personal devices, participants had to manually log screen time and later upload daily screenshots during assessment weeks, increasing burden and potential for error. While StandUPTV could be downloaded to Android phones, iOS access requires further permissions, which are planned for future studies. Reducing technology set-up burden and employing robust participant engagement strategies are critical to increasing retention. Several participants reported that they viewed the educational content only at the start; delivering weekly lessons may sustain engagement. Although StandUPTV is substantially larger than previous rSST interventions in adults, delays in study start and difficulties in recruitment meant that we did not achieve our initial recruitment goal of 240, which may have impacted our ability to detect changes across intervention versions for secondary outcomes. A predominately female sample limits generalizability.

This study has important strengths and directly addresses several research gaps from systematic reviews of sedentary behavior interventions among adults [21, 22, 44]. We included a broad age range that includes different generations (millennials through baby boomers) who have distinct rSST consumption habits [29]. We systematically and rigorously evaluated an efficacious mHealth intervention that requires minimal staff and participant interaction after randomization, enhancing potential scalability. This study was longer and included a larger sample than previous rSST studies, and our full-factorial design enabled the testing of individual and combined effects of components. In contrast to previous studies that relied on self-report questionnaires as the primary outcome measure, we used a novel objective measure that includes multiple components of rSST beyond just TV viewing, moving this research area into the modern era of rSST assessment.

StandUPTV offers key insights into future research directions. While this study lasted twice as long as prior rSST interventions, studies are needed to assess whether behavior change persists beyond 16 weeks. Future work should compare long-term outcomes of the two optimized interventions—EARN vs. EARN & LOCKOUT & TEXT—and explore individual differences in effectiveness and preference that may impact behavior change maintenance. For instance, some may benefit more from LOCKOUT’s immediate consequence for exceeding the weekly limit, while others may not need them. Some participants reported that the EARN ratio was too lenient, suggesting a need to test lower or personalized ratios (e.g., 2:1). Though TEXT’s impact was mixed, some participants valued its real-time reminders. Future research should also aim to test mechanisms of rSST change and the impact of reductions in rSST on other 24-hour behaviors, mental and physical health.

## Conclusions

StandUPTV is the first known study to systematically evaluate different strategies to reduce rSST among adults. In combination with a core intervention that included behavior change content, self-monitoring and a 50% reduction in rSST target, we found that intervention versions that produced the greatest reduction in rSST were EARN and EARN & LOCKOUT & TEXT. This study provides important evidence on efficacious multicomponent interventions that should be moved forward to the evaluation phase of the MOST framework to test the effect of rSST reductions on health outcomes.

## Electronic supplementary material

Below is the link to the electronic supplementary material.

Supplementary Material 1

## Data Availability

No datasets were generated or analysed during the current study.

## Abbreviations

MVPA: Moderate to vigorous physical activity
BMI: Body mass index
rSST: Recreational sedentary screen time
TV: Television viewing
