# Supplementary material for: StandUPTV: a full-factorial optimization trial to reduce sedentary screen time among adults
Source: Int J Behav Nutr Phys Act. 2025 Jun 13;22:77. doi: 10.1186/s12966-025-01771-2 (PMC12166558; doi:10.1186/s12966-025-01771-2)
Supplement: Supplementary file 1 — Supplementary Material 1 [file 12966_2025_1771_MOESM1_ESM.docx]

Supplemental Document

| **Supplemental Table 1: Primary behavior change techniques (Michie et al., 2013) used within core and experimental components** | | | | |
| --- | --- | --- | --- | --- |
|  | **CORE** | **EARN** | **LOCKOUT** | **TEXT** |
| Feedback on behavior | X |  |  |  |
| Self-monitoring of behavior | X |  |  |  |
| Instruction on performing the behavior | X |  |  |  |
| Information about health consequences | X |  |  |  |
| Goal setting (behavior) | X |  |  |  |
| Behavior substitution |  | X |  |  |
| Material Incentive (behavior) |  | X |  |  |
| Behavior Cost |  |  | X |  |
| Action planning |  |  | X |  |
| Social reward |  |  |  | X |
| Prompts/cues |  |  |  | X |
|  |  |  |  |  |

| **Supplemental Table 2. Baseline demographics and outcome measures by component level.** | | | | | | | | | | | | | | | |
| --- | --- | --- | --- | --- | --- | --- | --- | --- | --- | --- | --- | --- | --- | --- | --- |
| Experimental component | | Sample Size | Female No. (%) | Age M (SD) years | BMI Mean (SD) kg/m2 | Race, No. (%) | | | | Ethnicity, No. (%) | | rSST M (SD) min/day | Total sedentary time M (SD) min/day | MVPA  M (SD) min/day | Steps M (SD) steps/day |
|  |  |  |  |  |  | Caucasian | Black | Asian | Other | Not Hispanic | Hispanic |  |  |  |  |
| Overall |  | n=110 | 90 (81.8%) | 42.0 (11.7) | 29.7 (7.8) | 82 (74.6%) | 5 (4.6%) | 14 (12.7%) | 12 (10.9%) | 87 (79.1%) | 23 (20.9%) | 184.1 (125.8) | 641.4 (98.8) | 20.6 (14.8) | 6736.4 (2815.6) |
| LOCKOUT |  |  |  |  |  |  |  |  |  |  |  |  |  |  |  |
|  | On | n=55 | 46 (83.6%) | 42.3 (11.8) | 31.4 (9.1) | 41 (74.6%) | 3 (5.5%) | 6 (10.9%) | 7 (12.7%) | 44 (80.0%) | 11 (20.0%) | 193.5 (122.1) | 646.4 (105.3) | 19.9 (17.1) | 6409.0 (3022.7) |
|  | Off | n=55 | 44 (80.0%) | 41.7 (11.7) | 28.1 (5.9) | 41 (74.6%) | 2 (3.6%) | 8 (14.6%) | 5 (9.1%) | 43 (78.2%) | 12 (21.8%) | 175.5 (129.8) | 636.9 (93.2) | 21.1 (12.4) | 7037.1 (2606.0) |
| TEXT |  |  |  |  |  |  |  |  |  |  |  |  |  |  |  |
|  | On | n=59 | 47 (79.7%) | 41.6 (12.1) | 28.9 (6.3) | 41 (69.5%) | 3 (5.1%) | 8 (13.6%) | 6 (10.2%) | 46 (78.0%) | 13 (22.0%) | 167.4 (105.3) | 640.0 (104.6) | 22.2 (16.9) | 6741.7 (2950.7) |
|  | Off | n=51 | 43 (84.3%) | 42.4 (11.3) | 30.7 (9.3) | 41 (80.4%) | 2 (3.9%) | 6 (11.8%) | 6 (11.8%) | 41 (80.4%) | 10 (19.6%) | 205.8 (146.8) | 643.2 (92.0) | 18.5 (11.2) | 6729.7 (2666.9) |
| EARN |  |  |  |  |  |  |  |  |  |  |  |  |  |  |  |
|  | On | n=57 | 45 (79.0%) | 40.8 (11.8) | 28.8 (7.4) | 39 (68.4%) | 3 (5.3%) | 11 (19.3%) | 4 (7.0%) | 47 (82.5%) | 10 (17.5%) | 196.7 (123.6) | 643.7 (108.4) | 18.5 (12.3) | 6568.4 (2835.4) |
|  | Off | n=53 | 45 (84.9%) | 43.3 (11.5) | 30.8 (8.1) | 43 (81.1%) | 2 (3.8%) | 3 (5.7%) | 8 (15.1%) | 40 (75.5%) | 13 (24.5%) | 171.0 (128.1) | 639.0 (88.8) | 22.7 (16.8) | 6911.8 (2815.1) |

Note: rSST is recreational sedentary screen time, Sedentary time, Light physical activity and moderate physical activity were all assessed using the activPAL.

| **Supplemental Table 3: Baseline demographics and outcome measures between those who did and did-not complete the final assessment.** | | | | |
| --- | --- | --- | --- | --- |
|  |  | Overall | Completer | Non-completer |
|  | | n=110 | n= 80 | n= 30 |
| Female | No. (%) | 90 (81.8%) | 64 (80.0%) | 26 (86.7%) |
| Age | years | 42.0 (11.7) | 41.4 (11.2) | 43.7 (12.9) |
| BMI | Mean (SD) kg/m2 | 29.7 (7.8) | 29.5 (7.9) | 30.5 (7.7) |
| Race, No. (%) | Caucasian | 82 (74.6%) | 63 (78.8%) | 19 (63.3%) |
|  | Black | 5 (4.6%) | 2 (2.5%) | 3 (10.0%) |
|  | Asian | 14 (12.7%) | 9 (11.3%) | 5 (16.7%) |
|  | Other | 12 (10.9%) | 6 (12.5%) | 3 (10.0%) |
| Ethnicity, No. (%) | Non- Hispanic | 87 (79.%) | 62 (77.5%) | 25 (83.3%) |
|  | Hispanic | 23 (20.9%) | 18 (22.5%) | 5 (16.7%) |
| rSST | min/day | 184.1 (125.8) | 178.6 (120.1) | 194.8 (143.9) |
| Total sedentary time | min/day | 641.4 (98.8) | 642.1 (95.1) | 628.7 (100.5) |
| LPA | min/day | 68.7 (28.1) | 70.1 (30.4) | 64.4 (19.3) |
| MVPA | min/day | 20.6 (14.8) | 17.6 (14.6) | 19.6 (17.5) |
| Steps | steps/day | 6736 (2816) | 6812 (2879) | 6686 (2824) |

Note: rSST is recreational sedentary screen time, Sedentary time, Light physical activity and moderate physical activity were all assessed using the activPAL.

**Supplemental Table 4: Feasibility, User Burden, and Acceptability Outcomes Overall and by Experimental Condition**

| **Study feasibility** | | Overall | LOCKOUT+EARN+TEXT | LOCKOUT+EARN | LOCKOUT+TEXT | LOCKOUT | TEXT+EARN | EARN | TEXT | CORE ONLY |
| --- | --- | --- | --- | --- | --- | --- | --- | --- | --- | --- |
|  | Randomized (N) | 110 | 16 | 10 | 15 | 14 | 15 | 16 | 13 | 11 |
|  | Study-related withdraw (N[%]) | 11 (10%) | 0 (0%) | 3 (30%) | 2 (13.3%) | 2 (14.3%) | 1 (6.7%) | 0 (0%) | 2 (15.4%) | 1 (9.1%) |
|  | Non-study related withdraw (N[%]) | 5 (4.5%) | 1 (6.3%) | 0 (0%) | 0 (0%) | 1 (7.1%) | 0 (0%) | 1 (6.3%) | 2 (15.4%) | 0 (0%) |
|  | Missing 16-week assessment (N[%]) | 14 (12.7%) | 1 (6.3%) | 2 (20%) | 5 (33%) | 1 (7.1%) | 1 (6.7%) | 2 (12.5%) | 0 (0%) | 2 (18.2%) |
|  | Completed 16-week assessment (N[%]) | 80 (72.8%) | 14 (87.5%) | 5 (50%) | 8 (53.3%) | 10 (71.4%) | 13 (86.7%) | 13 (81.3%) | 9 (69.2%) | 8 (72.7%) |
| **Acceptability** | | **% satisfied/very satisfied or % yes** | | | | | | |  |  |
|  | Overall experience as a study participant (% satisfied) | 87.1 | 100.0 | 100.0 | 100.0 | 87.5 | 75.0 | 91.7 | 66.7 | 85.7 |
|  | Overall Satisfaction with StandupTV app (% satisfied) | 58.1 | 54.5 | 50.0 | 66.7 | 75.0 | 37.5 | 66.7 | 55.6 | 57.2 |
|  | Overall, was the StandUPTV app helpful in reducing your recreational sedentary screen time? (% yes) | 93.4 | 100.0 | 100.0 | 100.0 | 100.0 | 87.5 | 100.0 | 77.8 | 85.7 |
|  | If it were made available, would you be interested in continuing the use of the StandUPTV app after the study is over? (% yes) | 78.7 | 81.8 | 50.0 | 100.0 | 75.0 | 75.0 | 81.8 | 77.8 | 85.7 |
|  | Would you download an app like this to your smartphone? (% yes) | 83.9 | 81.8 | 50.0 | 66.7 | 87.5 | 75.0 | 91.7 | 100.0 | 85.7 |
|  | Would you recommend the StandUPTV app to other people? (% yes) | 91.8 | 90.9 | 75.0 | 100.0 | 100.0 | 87.5 | 91.7 | 87.5 | 100.0 |
|  | Proportion using app at least 1/day (% yes) | 60.7 | 72.7 | 100.0 | 33.3 | 75.0 | 57.1 | 41.7 | 66.7 | 42.9 |
|  | Satisfaction with technical support | 84.8 | 90.9 | 75.0 | 66.7 | 87.5 | 62.5 | 91.7 | 88.9 | 100.0 |
|  | Responsiveness of study staff | 100 | 100 | 100 | 100 | 100 | 100 | 100 | 100 | 100 |
| **User burden** | |  |  |  |  |  |  |  |  |  |
|  | Total Score, M(SD) | 7.2 (6.5) | 10.4 (7.9) | 6.0 (4.7) | 7.3 (6.0) | 4.7 (2.8) | 8.7 (6.0) | 5.3 (4.0) | 4.6 (4.5) | 9.1 (11.8) |
|  | Difficulty of Use, M(SD) | 0.8 (0.6) | 1.2 (0.7) | 0.5 (0.3) | 0.6 (0.5) | 0.7 (0.4) | 0.9 (0.6) | 0.6 (0.6) | 0.6 (0.6) | 0.9 (1.0) |
|  | Physical, M(SD) | 0.1 (0.2) | 0.1 (0.3) | 0.0 (0.0) | 0.0 (0.0) | 0.0 (0.0) | 0.0 (0.0) | 0.0 (0.0) | 0.0 (0.0) | 0.0 (0.0) |
|  | Time and Social, M(SD) | 0.3 (0.4) | 0.6 (0.7) | 0.3 (0.3) | 0.4 (0.5) | 0.2 (0.2) | 0.5 (0.5) | 0.3 (0.5) | 0.3 (0.3) | 0.5 (1.1) |
|  | Mental and Emotional, M(SD) | 0.4 (0.6) | 0.0 (0.1) | 0.0 (0.0) | 0.2 (0.3) | 0.1 (0.3) | 0.1 (0.2) | 0.1 (0.1) | 0.0 (0.0) | 0.1 (0.2) |
|  | Privacy, M(SD) | 0.3 (0.6) | 0.3 (0.5) | 0.6 (1.0) | 0.5 (1.0) | 0.1 (0.1) | 0.5 (0.8) | 0.1 (0.2) | 0.1 (0.2) | 0.5 (0.8) |
|  | Financial, M(SD) | 0.01 (0.1) | 0.4 (0.6) | 0.3 (0.2) | 0.4 (0.5) | 0.2 (0.3) | 0.4 (0.4) | 0.3 (0.3) | 0.2 (0.3) | 0.4 (0.5) |

| **Supplementary Table 5. Additional Satisfaction with component-specific app features** | |  |  |  |
| --- | --- | --- | --- | --- |
|  |  | Not at all | Mostly | Very |
| Total Sample (N=64) | Overall, how satisfied were you with the StandUPTV app? | 12.5% | 39.1% | 48.4% |
| TEXT (n=32) | Overall, how satisfied were you with the StandUPTV app? | 18.8% | 37.5% | 43.8% |
|  | How helpful was the content of the text messages? | 25.0% | 40.6% | 34.4% |
|  | How helpful was the frequency of text messages? | 37.5% | 31.3% | 31.3% |
|  | How helpful were the text messages in changing your behavior? | 53.1% | 28.1% | 18.8% |
| EARN (n=36) | Overall, how satisfied were you with the StandUPTV app? | 5.6% | 41.7% | 52.8% |
|  | How helpful was the opportunity to earn screen time through exercise in reducing your screen time? | 8.3% | 22.2% | 69.4% |
|  | How satisfied are you with the feedback on your exercise? | 16.7% | 25.0% | 55.6% |
|  | How satisfied are you with the planning tool to track your exercise? | 50.0% | 38.9% | 11.1% |
| LOCKOUT (n=26) | Overall, how satisfied were you with the StandUPTV app? | 3.7% | 51.9% | 44.4% |
|  | Was the planning tool helpful in helping you stay within your screen time goal? | 53.8% | 38.5% | 7.7% |
|  |  |  |  |  |
| Note: Scores ranged from 0 to 10, with scored < 3 considered "not at all", 3-7 were "mostly" and 8-10 were "very" | |  |  |  |

| **Supplementary Table 6: Main themes and example responses from open-ended exit interview questions** | |
| --- | --- |
| **Main themes** | **Example responses** |
| What did you like best about the app? (Gague) | “I think that now a days we are glued to our phones. I wanted to change that. If you get sucked in for hours. Giving yourself a time budget. Seeing the meter. The visual aspect was SUPER helpful.” |
|  | Liked the gauge that showed how much time used / how much time had left - really controlled as she got toward the end. Engaged in other habits, picking up a book. |
| Theme: Increases awareness of screen time | Aware of my screen time more than before; knowing data was being collected made me take breaks before turning it back on. |
|  | How much free time I actually have and making an attempt to use it better. |
|  | Yes - I definitely learned how little bouts of watching and sitting around idly adds up throughout the day - how innate and automatic behavior sedentary screen time is. Feels that accepts sedentary screen time as a reality of life. More significantly. |
| Feedback on the Educational Content? | The tips were good at the beginning, but as the study went on I didn't really follow through with them. |
|  | One thing that stood out was the mindfulness piece. It was a good reminder. For example, if I wanted to watch TV, I would pull out my yoga mat and stretch out while I watched TV. Social support tip was helpful in setting a buddy system for working out |
| What did you like least? | Not being able to access log of notifications. I couldn't access a previous notification until a new one came through. |
|  | Didn't like the android platform. Tablet was clunky. |
|  | Sometimes it took a while to load. It might have been the tablet. Wasn't clear how to navigate through some of the syncing. |
|  | I have all good things to say. I wish I could have used my phone instead of the tablet. I think that would have been easier to participate. |
|  | The ongoing bouts, it happened several times when TV was off. She wasn't able to edit the bouts |
| Feedback on the TEXT component? | It seemed like they were general tips and not targeted to me. They need to be targeted to what the person is doing. The ones that provided action were really good but I would get them randomly. They didn't seem related to what I was actually doing. |
|  | They would come all at once, I would recommends a text notification to personal phone in addition to the notifications via tablet. |
|  | I think it's because I was mostly working a bunch so there would be like 6 of them and I would just swipe away. They were timed for when I was just getting on the app rather than when I had been on it a lot. |
| Feedback on the EARN condition? | Narrowing the ratio may solicit the desired behavior. Maybe allowing participants to select their ratio would have been good.. I think this would help people push themselves a bit more. |
|  | Being in the EARN was definitely a good incentive. |
| Feedback on the LOCKOUT condition? | might have been nice to have a notification that would alert you about the percentage of screen time you've gotten to, or a countdown of the time that you have left for the week / day |
|  | I thought my TV broke so I reset the Wemo. I realized later that was actually the lock out. |
